# Tim-3 Negatively Regulates Cytotoxicity in Exhausted CD8^+^ T Cells in HIV Infection

**DOI:** 10.1371/journal.pone.0040146

**Published:** 2012-07-05

**Authors:** Ali Sakhdari, Shariq Mujib, Bahareh Vali, Feng Yun Yue, Sonya MacParland, Kiera Clayton, Richard Bradley Jones, Jun Liu, Erika Yue Lee, Erika Benko, Colin Kovacs, Jennifer Gommerman, Rupert Kaul, Mario A. Ostrowski

**Affiliations:** 1 Department of Immunology, University of Toronto, Toronto, Ontario, Canada; 2 Institute of Medical Science, University of Toronto, Toronto, Ontario, Canada; 3 Maple Leaf Medical Clinic, Toronto, Canada; 4 Li Ka Shing Knowledge Institute of St. Michael’s Hospital, Toronto, Canada; Massachusetts General Hospital, United States of America

## Abstract

Cytotoxic CD8^+^ T cells (CTLs) contain virus infections through the release of granules containing both perforin and granzymes. T cell ‘exhaustion’ is a hallmark of chronic persistent viral infections including HIV. The inhibitory regulatory molecule, T cell Immunoglobulin and Mucin domain containing 3 (Tim-3) is induced on HIV-specific T cells in chronic progressive infection. These Tim-3 expressing T cells are dysfunctional in terms of their capacities to proliferate or to produce cytokines. In this study, we evaluated the effect of Tim-3 expression on the cytotoxic capabilities of CD8^+^ T cells in the context of HIV infection. We investigated the cytotoxic capacity of Tim-3 expressing T cells by examining 1) the ability of Tim-3^+^ CD8^+^ T cells to make perforin and 2) the direct ability of Tim-3^+^ CD8^+^ T cells to kill autologous HIV infected CD4^+^ target cells. Surprisingly, Tim-3^+^ CD8^+^ T cells maintain higher levels of perforin, which was mainly in a granule-associated (stored) conformation, as well as express high levels of T-bet. However, these cells were also defective in their ability to degranulate. Blocking the Tim-3 signalling pathway enhanced the cytotoxic capabilities of HIV specific CD8^+^ T cells from chronic progressors by increasing; a) their degranulation capacity, b) their ability to release perforin, c) their ability to target activated granzyme B to HIV antigen expressing CD4^+^ T cells and d) their ability to suppress HIV infection of CD4^+^ T cells. In this latter effect, blocking the Tim-3 pathway enhances the cytotoxcity of CD8^+^ T cells from chronic progressors to the level very close to that of T cells from viral controllers. Thus, the Tim-3 receptor, in addition to acting as a terminator for cytokine producing and proliferative functions of CTLs, can also down-regulate the CD8^+^ T cell cytotoxic function through inhibition of degranulation and perforin and granzyme secretion.

## Introduction

The inability of T cell-mediated immune responses to control persistent viral infections, like human immunodeficiency virus-1 (HIV), has been correlated with the impairment in the ability of virus-specific T cells to produce cytokines, to proliferate and to survive [1], [2]. This dysfunction, referred to as ‘T cell exhaustion’ in the setting of HIV infection, allows for continuing viral replication in most of the infected individuals and the inexorable progression to AIDS [1], [3], [4], [5], [6], [7], [8], [9], [10], [11], [12], [13]. T cell exhaustion was first described in the lymphocytic choriomeningitis virus (LCMV)-infected mice, in which certain LCMV strains induced virus-specific effector CD8^+^ T cells that failed to produce effector cytokines upon antigen stimulation [14]. We previously identified a novel population of ‘exhausted’ T cells in HIV infected individuals, which are marked by increased surface expression of the glycoprotein Tim-3. These cells, in contrast to programmed cell death -1 (PD-1) expressing cells, are relatively more deficient in effector cytokine production [15]. Tim-3 expression was shown to be upregulated on HIV specific CD8^+^ T cells [15]. More notably, blocking the Tim-3 signaling pathway *in vitro* restored proliferation and enhanced cytokine production in HIV-specific T cells [15]. It has recently been shown that Tim-3 expression is dependent on the CD4^+^ Th1 and CD8^+^ Tc1 transcription factor T-bet [16]. This transcription factor is also required for proper perforin production and function in cytotoxic lymphocytes [16], [17], [18], [19].

Cytotoxic CD8^+^ T lymphocytes (CTLs) kill their virally infected or transformed target cells predominantly through the release of lytic substances, mainly perforin and granzymes, which are secreted via exocytosis of pre-formed granules [20], [21], [22], [23]. There is little question regarding the crucial importance of perforin in the control of infectious pathogens. Indeed, mutation or dysregulation of perforin in humans results in compromised cellular immunity and enhanced susceptibility to viral infections [24]. Granule-mediated killing by CD8^+^ T cells occurs within minutes of target cell recognition [25], [26], [27]. Recently, another mechanism for perforin replenishment has been identified which is the rapid upregulation and targeted release of newly-produced perforin, which traffics to the immunological synapse via a route that largely bypasses cytotoxic granules [28]. This *de novo* synthesis of perforin by CD8^+^ T cells can be easily detected by flow cytometry in conjunction with standard intracellular cytokine-staining (ICS) [29].

While many cell surface markers, activation profiles, and functional parameters of both *ex vivo* HIV-specific CD8^+^ and CD4^+^ T cells have been shown to correlate with control of viremia [8], [30], [31], [32], [33] few, if any, can potentially mediate direct control of HIV replication through the lysis of infected cells [34]. Our lab has shown that Tim-3 expressing CD8^+^ T cells are dysfunctional in terms of polyfunctionality, proliferative ability, cytokine release and inhibitory receptor expression [15]. Here we examined the *ex vivo* cytotoxicity of Tim-3 expressing CD8^+^ T cells by examining their perforin content, ability to degranulate [35], [36] and also through direct measurement of cytotoxicity [37].

## Materials and Methods

### Ethics Statement

Informed consent was obtained in accordance with the guidelines for conduction of clinical research at the University of Toronto and Maple Leaf Clinic institutional ethics boards. Written Informed Consent was provided for this study, which was reviewed by research ethics board of the University of Toronto, Canada and of St. Michael’s Hospital, Toronto, Canada.

### Patient Groups

Our cohort consists of two different patient groups including:

1) Chronic progressive HIV infection (*CP*) (HIV infection >1 year, with active viral replication, i.e., detectable viremia >10,000 bDNA copies/ml for at least one year (25 patients) and 2) Viral controllers (VC) defined as asymptomatic, untreated HIV infection for at least 2 years with no consistent decline in peripheral blood CD4 count, and low or undetectable levels of plasma viremia (less than 500 copies/ml, bDNA assay) (n = 7, mean VL = 125 copies/ml, mean CD4 count = 973/ mm^3^). We also studied three HCV mono-infected individuals and three CMV sero-positive, HIV-negative, HCV-negative individuals.

### Flow Cytometry

Peripheral Blood Mononuclear Cells (PBMC) were incubated with cognate antigen at 2 µg/ml/peptide for 6 h in the presence of Brefeldin A and Monensin and 1 µg/ml of anti-CD49d and anti-CD28 Ab for co-stimulation (BD Biosciences, San Jose, CA, USA) and stained for CD107a FITC or APC (BD). Cells were then washed, permeabilized, and stained with respective antibodies: IFN-γ–PE or Alexa 647, PD-1–FITC or APC, Tim-3–PE or APC, Perforin (clone B-D48)–PerCp (from Abcam, Cambridge, MA), Perforin (clone δG9)-FITC (from Abcam), CD3-APC-Cy7 or APC or FITC, CD45RA-PE-Cy7, CD8-PE or FITC or Alexa 700 or PE-TR, CD4-PerCP or APC or PercP-cy7, anti-HIV Gag p24-PE (Kc57 RDI) and respective isotype controls. Antibody to T-bet was from BD Biosciences. Cells were fixed in 1% paraformaldehyde/PBS and then analyzed on a FACSCalibur or LSR II (BD Biosciences), and data were acquired by CellQuest software (BD) and analyzed with FlowJo (TreeStar, San Carlos, CA). Between 10.000 and 200.000 events in the lymphocyte gate were acquired per sample. Overlapping HIV Clade B Gag pooled peptides were obtained from the National Institutes of Health AIDS Research and Reference Reagent Program.

### Perforin ELISA

Frozen PBMC were thawed and CD8^+^ T cells were sorted using negative selection (CD8^+^ T cell Isolation Kit II, Miltenyi Biotec) and plated at 2 × 10^5^ cells in 200 µl complete R10 medium (RPMI 1640+10% FBS+1 U/ml penicillin+100 µg/ml streptomycin +2 mM glutamine +20 mM HEPES) and stimulated with a pool of overlapping HIV Gag peptides (2 µg/ml/peptide) or a pool of 10 HCV NS3 peptides (10 µg/ml/peptide) or a pool of overlapping CMV pp65 peptides (2 µg/ml/peptide) or anti-CD3/CD28 (final concentration 1 µg/ml) or media alone. An antagonistic anti-Tim-3 antibody (2E2) [15] or a corresponding isotype antibody (IgG1) was used to compare the effects of Tim-3 pathway blocking on perforin release. Supernatants were harvested after 6 h. Perforin levels were detected using the Perforin Human ELISA Kit-ab46115 (Abcam, Cambridge, MA), according to the manufacturer’s specifications.

### HIV Gag mRNA Transfection

The HIV *gag* template DNA is taken from a plasmid encoding for HIV Gag from the NIH AIDS reagent program. Briefly, we PCR-amplified HIV *gag* from the provided plasmid using restriction enzyme sites HindIII on the 5′ and EcoRI on the 3′ ends. The PCR product was cloned into the vector pGEM4Z/GFP/A64. This vector basically encodes GFP with 3′ 64-adenine tail. The GFP coding sequence was excised and replaced with a codon-optimized HIV *gag* DNA. Vector was grown in bacteria (E. Coli) and maxi-prepped to get DNA. The enzyme Spel was used for linearization. The linear vector was then used in Ambion Inc’s T7 mMessage mMachine kit. mRNA was purified using Megaclear (Ambion). mRNA was diluted to a concentration of 2 µg/µL. 2 µL of diluted mRNA was used for transfection by electroporation. Transfection efficacy ranged from 25% to 45%. However since our comparison was intra-subject and not inter-subject we were still able to use different efficacies.

**Figure 1:**
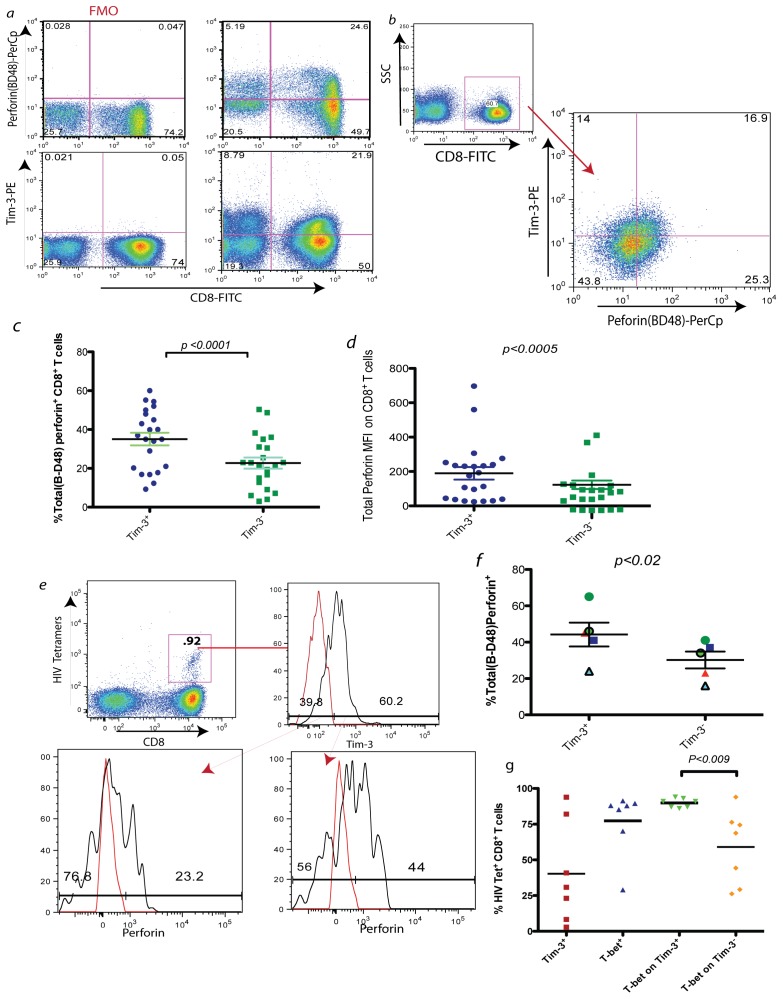
Total and antigen specific Tim-3^+^ CD8^+^ T cells have higher frequencies of perforin expression. *Ex vivo* PBMC from untreated chronically HIV infected subjects were stained for perforin (clone B-D48) and Tim-3. In *a),* shown are FMO staining for Tim-3, and B-D48 perforin antibodies of a representative sample, in *b),* representative gating strategy for Tim-3, and B-D48 perforin and a representative experiment showing the percentage of perforin and Tim-3 expressions on total CD8^+^ CD3^+^ T cells. Summary of data for 22 individuals showing perforin content of Tim-3^+^ and Tim-3**^−^** CD8^+^ T cells as a % in *c*) and MFI in *d*). In *e*) a representative experiment where *ex vivo* CD8^+^ T cells are stained with a pool of HIV specific tetramers (see Methods) and perforin expression on gated Tim-3^+^ and Tim-3**^−^** T cells are shown, with a summary shown in *f*) for 5 chronically infected individuals. Each symbol represents a single individual. Red histogram represents FMO. In *g*) *Ex vivo* PBMC from 7 chronic progressors were stained for HIV-specific tetramers and examined for Tim-3 and T-bet expression. The individual in *e)* was not examined in *g*) (*p* value based on two tailed paired t test).

### Granzyme B Cytotoxicity Assay

2×10^6^ transfected (with HIV *gag* mRNA) CD4^+^ T cells were labeled with either TFL-4 or NFL-1 or both for 15 mins (as per manufacturer instructions-GranToxiLux, OncoImmunin, Gaithersburg, MD, USA) [38]. Negatively selected effector CD8^+^ T cells (of the same sample) that were incubated for one day with blocking 2E2 anti-Tim-3 Ab (10 µg/ml) or isotype IgG1 (10 µg/ml) or media alone were then added to labeled target cells in different ratios (3∶1,1∶1,1∶3), for 1 hr. At the beginning of the co-incubation the effector/target cells were washed and a Granzyme B substrate was added to the wells. The cytotoxicity of the cells was compared by measuring the number of killed target cells (positive for cleaved Granzyme B substrate) at each ratio and in different conditions. The GranToxiLux killing assay was conducted per manufacturer’s protocols (OncoImmunin) except where otherwise noted.

### HIV Infection of Target Cells

Virus production: CD4^+^ T cells from an HIV negative donor were activated with anti-CD3/28 and 50 U/mL IL-2 in R-10 media for 48 h. The primary HIV isolate 91US-1 (obtained from NIH AIDS Research and Reference Reagent Program) was then added at an m.o.i of 0.2 at a cell concentration of 4–10 x10^6/^ml. The infection mixture was incubated for 4 days at 37°C. The infection was monitored with intracellular HIV p24 staining daily to detect the peak of infection (ranges from 40–90% p24^+^ cells) at which point cells were pelleted down at 300×g for 10 min and supernatant was collected. The virus was then sucrose purified prior to magnetofection. Briefly, a 500 µL aliquot of virus was layered on top of 100 µL of 20% Sucrose solution +5 mM EDTA in PBS. Tubes were centrifuged at 35000×g for 45 min. We then discarded all but 20 µl of the supernatant, which contained the virus pellet.

Virus infection of target cells: We added OZ BioSciences ViroMag beads to bind HIV virus particles made above. Virus was then added to gently pelleted target CD4^+^ T cells on a plate of target cells (20×g for 2 min, just to bring the cells to the bottom of the plate). The plate was then kept on a magnet in a 37°C incubator for 3 hours. The plate was removed from the magnet and infection was monitored on a daily basis by HIV Gag p24 intracellular staining. The infection efficacy ranged between 25 to 60%. We also used sucrose-purified culture media alone (e.g. R-10+50U/ml IL-2) as the negative control infection condition.

### Infected CD4^+^ T Cell Suppression Assay

In assays using CD4^+^ T cell targets infected with US1/GS 004 (91US-1), target cells were co-incubated with CD8^+^ T cells from the same sample (in the presence of 2E2 Ab or isotype) in different effector: target (E: T) ratios (10∶1,1∶1,1∶4,1∶16) for 3 days. Cells were then treated with Cytofix/Cytoperm (BD Biosciences, San Jose, CA, USA) prior to staining for confirmation of infection and measurement of elimination of gag p24-expressing cells. The truly infected target cell numbers were measured on the basis of the total percentages of p24^+^ cells in CD4^+^ T cells without CD8^+^ T cells at day 3. The number of remaining HIV infected target cells (p24^+^) was measured and compared between two different groups.

### Tetramer Staining

The following tetramer pools to HIV (HLA-A*0201-SLYNTVATL[Gag], HLA-B*0801-FLKEKGGL[Nef], HLA-B*0702-TPGPGVRYRL[Nef], HLA-B*0801-GEIYKRWII[Gag], HLA*0201-ILKEPVHGV[Pol]), or to CMV (HLA-B*0801-ELRRKMMYM, HLA-A*0201-NLVPMVATV, HLA-B*0702-TPRVTGGGAM, all from pp65) (Beckman Coulter, Fullerton, CA) were used for staining. 5×10^5^ to 1×10^6^ PBMC were stained with the pool of tetramers in 1 ml of 2% FBS in PBS with 2 mM EDTA for 20 minutes at 4°C, followed by staining for CD3 (BD Biosciences, Sandiego, CA), CD8 (BD), Tim-3 (MAb, R&D Systems, Minneapolis, MN), and perforin (B-D48-Abcam, Cambridge, MA) or T bet (BD, Sandiego, CA). At least 200,000 events were obtained using either an LSR II flow cytometer, or a FACSCalibur instrument (BD Biosciences, Sandiego, CA). Further analysis was performed using FlowJo version 7.6 (Tree Star Inc.).

### Statistical Analysis

To determine whether two groups were statistically different for a given variable, we used the Wilcoxon rank sum test (two-tailed) or the paired Student *t* test in Graphpad Prism version 5.00 (GraphPad software,La Jolla, CA).

**Figure 2:**
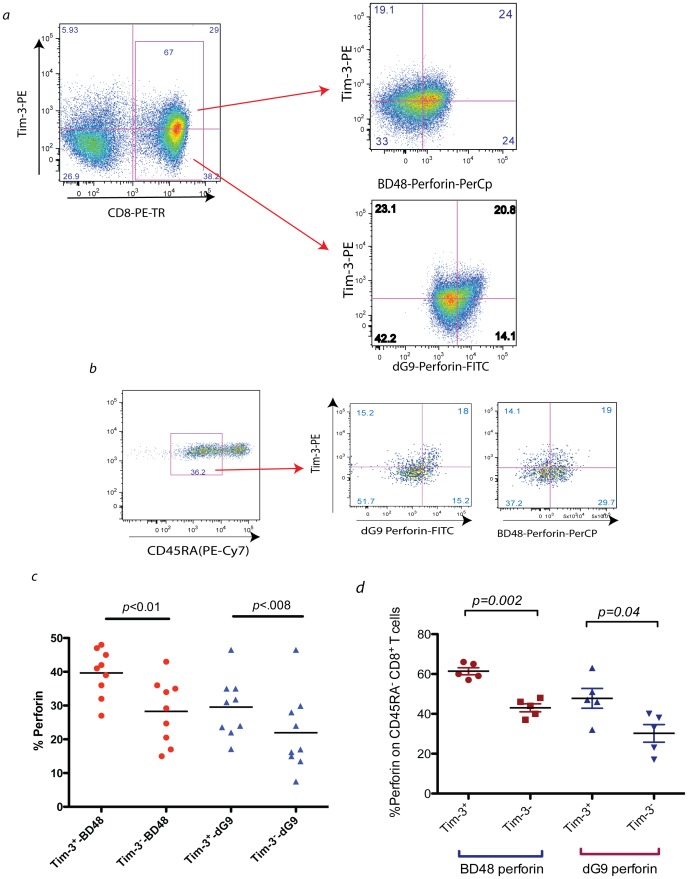
Perforin content of Tim-3^+^ CD8^+^ T cells as determined by two antibody clones. In *a*), a representative experiment showing Tim-3 expression on *ex vivo* CD8^+^ T cells from a treatment naïve chronically HIV infected subject showing perforin expression by two antibodies detecting different conformations of perforin on Tim-3^+^ or Tim-3**^−^**CD8^+^ T cells. The δG9 clone has been proposed to predominantly detect stored (granule associated) perforin and clone B-D48 detects stored perforin plus majority of other perforin conformations [23], [29]. In *b*) a representative figure showing the relationship between two different conformations of perforin and Tim-3 expressions after gating out the naïve and terminally differentiated CD8^+^ T cells by gating in all CD45RA^-^ memory subsets. In *c),* summary of data for all 9 chronically HIV infected individuals stratifying perforin antibodies and Tim-3 expression on total CD8^+^ T cells. In *d*) summary of data for 5 chronically HIV infected individuals showing the perforin expression with two clones and Tim-3 expressions of memory subsets of CD8^+^ T cells (*p* value based on two tailed paired t test).

## Results

### Tim-3^+^ CD8^+^ T cells express more perforin than their Tim-3^−^ counterpart in HIV infection

We previously showed that Tim-3 expression increases on CD8^+^ T cells in the context of HIV infection [15]. Tim-3 expressing CD8^+^ T cells have lower cytokine (IFN-γ or TNF-α) production or proliferative abilities compared to Tim-3 negative cells (data not shown) [15]. However, none of these functions represent the true cytotoxic activities of CD8^+^ T cells [23], [28]. We initially examined the perforin content of *ex vivo* CD8*^+^* T cells by flow cytometry from 22 treatment-naïve chronically HIV infected subjects (median CD4 count  = 455/µl, median HIV viral load  = 52,325 copies/ml) and compared the percentage of perforin expression in Tim-3 positive or negative CD8*^+^* T cells (Fig. 1). Two perforin specific monoclonal antibodies have characterized perforin in human CD8*^+^* T cells [28], [29]. The ∂G9 clone is limited to detecting the form of perforin found in the acidic milieu of the granules or associated with serglycin, thus predominantly detecting perforin stored in granules. The B-D48 clone, on the other hand, is able to recognize both the late granule associated form of perforin as well as its newly synthesized non-granule associated form. Thus, the B-D48 clone detects multiple conformations of perforin. Using the B-D48 clone, we found that Tim-3 expressing CD8*^+^* T cells express significantly more perforin than that of Tim-3 negative CD8*^+^* T cells (Fig 1 *c,d p*<0.0005). A representative experiment is shown in Fig. 1*b* and summary of all individuals in Fig. 1*c and 1d*(frequency of perforin expressing cells on total CD8^+^ T cells is 35.08%±15.06% for Tim-3^+^ vs. 22.70% ±13.47% for Tim-3**^−^**, mean ± SD, *p*<0.0001).

**Figure 3:**
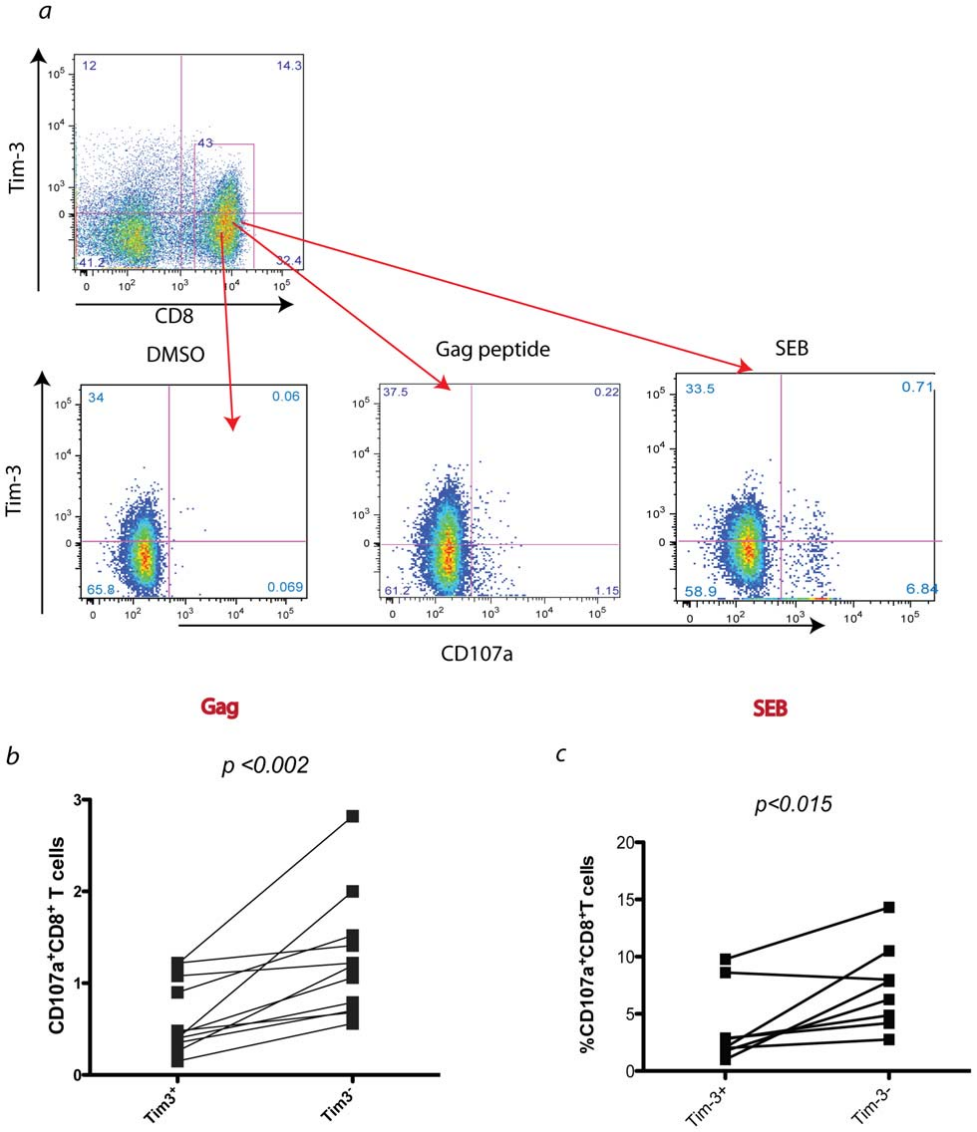
Tim-3^+^ CD8^+^ T cells cannot degranulate as effectively as Tim-3^−^ CD8^+^ T cells. *Ex vivo* PBMC from HIV chronically infected subjects were stimulated for 6 hours with a pool of HIV Gag peptides or SEB or DMSO and stained for CD107a and Tim-3. In *a), a* representative experiment showing higher CD107a expression in Tim-3 negative component of CD8^+^ T cells after stimulation with either pool of HIV antigens or SEB. In *b*), summary of all data for 10 chronically HIV infected individuals showing higher ability for degranulation in Tim-3 negative subpopulation of CD8^+^ T cells after stimulation with Gag peptides. In *c*) summary of all data for 8 chronically HIV infected individuals showing higher ability for degranulation in Tim-3 negative subpopulation of CD8^+^ T cells after stimulation with SEB (*p* value based on wilcoxon signed rank test).

### The Frequency of Perforin-expressing Tim-3^+^ HIV Specific T Cells is Higher than those of their Tim-3**^−^** Counterpart

Perforin expression (B-D48 clone) of antigen-specific CD8^+^ T cells was examined with HIV tetramer staining. For tetramer staining, we examined PBMC from 5 HLA-A02, HLA-B07 or HLA-B08 positive untreated chronically HIV infected individuals. We monitored antigen-specific CD8^+^ T cells by employing a pool of 5 MHC class I matched tetramers (see Methods) to determine the level of perforin on *ex vivo* CD8^+^ T cells. As is shown in Figure 1*e*, Tim-3 expressing HIV specific CD8^+^ T cells have higher frequencies of perforin positive cells than the Tim-3 negative tetramer staining population (Fig. 1*f*, mean = 44.20%±14.62% vs. 30.20%±10.38%, for Tim-3^+^ and Tim**^−^**3- cells respectively *p* = 0.022).

**Figure 4:**
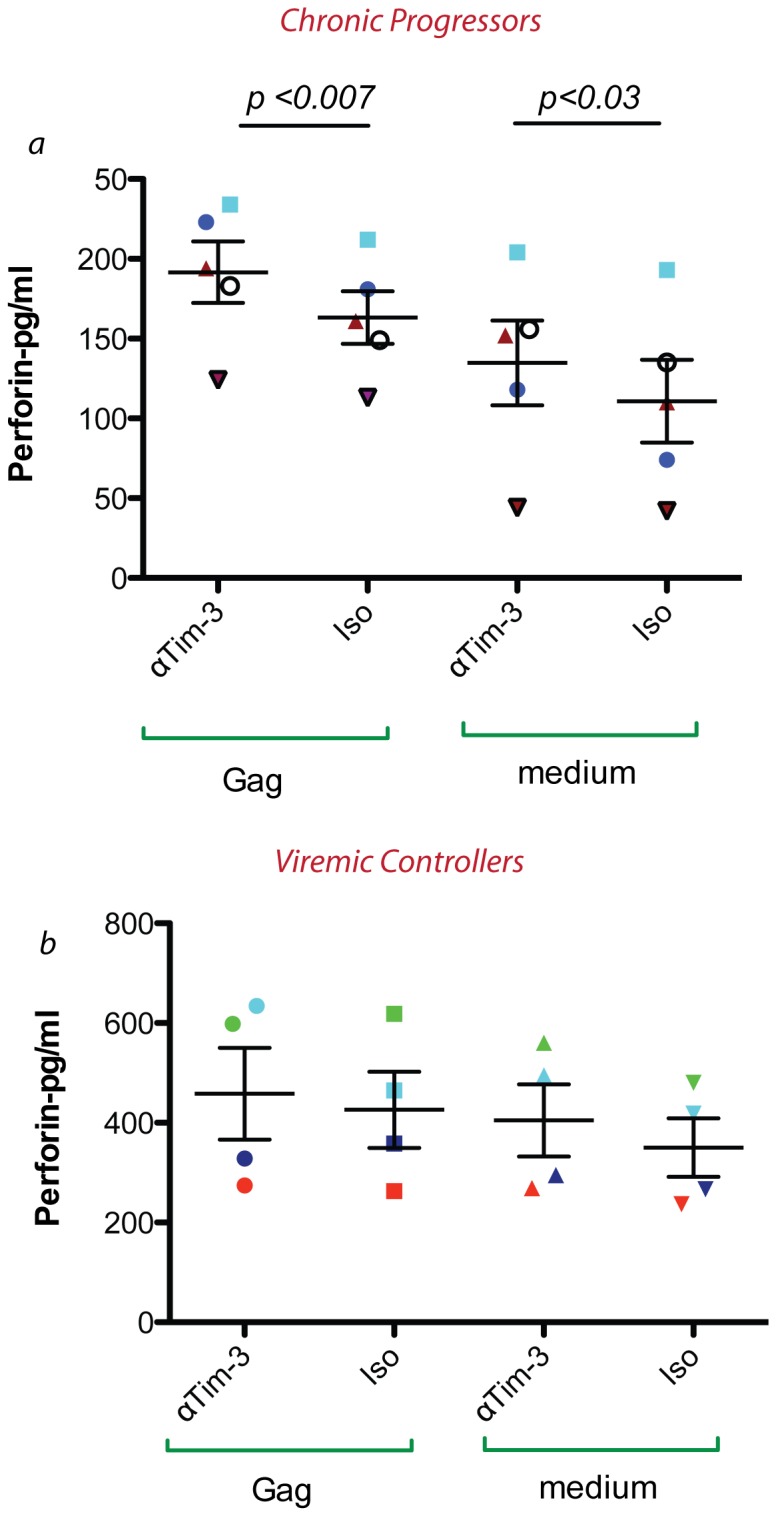
Tim-3 pathway blocking improves the perforin release in antigen specific CD8^+^ T cells only in chronically HIV infected individuals. 2×10^5^ negatively sorted CD8^+^ T cells from *ex vivo* PBMC from 5 untreated chronically infected (Fig *a*) and four untreated viral controllers (Fig *b)* were stimulated with a pool of HIV Gag peptides (final concentration of 2 µg/ml/peptide) for 6 h. Perforin released in supernatant was measured in an ELISA experiment in pg/ml. (αTim-3  =  2E2 Tim-3 blocking antibody, Cntr  =  IgG1 isotype control antibody) (*p* value based on two tailed paired t test).

### Tim-3 Expressing HIV Specific CD8^+^ T cells Express High Amounts of T-bet

The transcription factor, T-bet, is required for perforin synthesis [16], [17], [18], [19]. We thus examined the levels of T-bet expression on HIV specific CD8^+^ T cells in association with Tim-3. We observed that HIV specific T cells generally tended to express high levels of T-bet, however, gating on Tim-3 expressing HIV specific cells showed uniformly high expression of T-bet (Fig. 1*g*). As is shown in Fig 1*g*, T-bet expression is significantly higher in Tim-3^+^ CD8^+^ T cells than Tim-3**^−^** populations as is expected [16].

**Figure 5:**
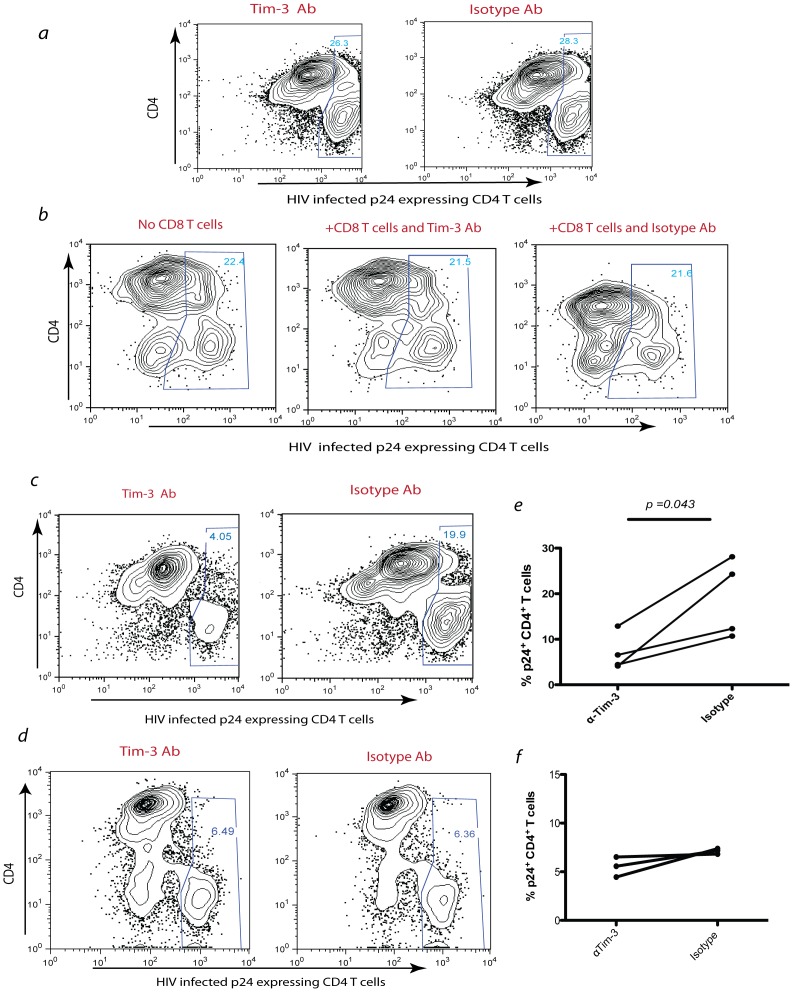
Tim-3 pathway blocking increases the antigen specific CD8^+^ T cells cytotoxicity and HIV suppression in chronic HIV infection. The effect of HIV Gag-specific CD8^+^ T cells after blocking the Tim-3 pathway with a Tim-3 blocking antibody (clone 2E2) on eliminating HIV-infected CD4^+^ T cells was tested in a virus suppression assay. Autologous CD4^+^ T cells (targets) are infected with a primary HIV virus isolate. Autologous CD8^+^ T cells (effectors) are added in 1∶1 ratio at the time of infection with 2E2 antagonistic anti-Tim-3 antibody or isotype at 10 µg/ml. The co-culture is incubated at 37°C for three days. The final readout is the percentage of HIV (p24^+^) positive target cells on day three examined by intracellular flow cytometry. In *a*) are autologous CD4^+^ T cells in the absence of autologous CD8^+^ T cells taken from an HIV infected individual after exogenous infection by HIV in the presence of Tim-3 antibody or isotype. Tim-3 blockade had no effect on total and infected CD4^+^ numbers. In *b*) shown are CD4^+^ T cells from an HIV uninfected normal volunteer infected with exogenous HIV and co-cultured with autologous CD8^+^ T cells (1∶1 ratio). Again we could not appreciate any difference in survival of CD4^+^ T cells. Tim-3 blockade had no effect on CD8^+^ mediated suppression of HIV Infection. In *c*), is a representative experiment showing the percentage of infected p24^+^ CD4^+^ T cells in the two different conditions in a chronic HIV infected individual and in *d)* is a representative experiment showing the percentage of infected p24^+^ CD4^+^ T cells in the two different conditions in a viral controller, Shown in *e*), are summary data for four chronically HIV infected individuals and in *f*) are summary data for three viral controllers. Each solid circle represents the average of three independent experiments from each individual showing the percentage of p24^+^ CD4^+^ T cells (*p* value based on two tailed paired t test).

### Tim-3^+^ CD8^+^ T Cells Express More Granule-associated Perforin than their Tim-3**^−^** Counterpart in HIV Infection

In order to further define the conformation of perforin that is expressed on Tim-3 positive CD8^+^ T cells, we also employed the anti-perforin antibody clone (δG9) that putatively detects the granule-associated ‘preformed or stored’ form of perforin [29]. We profiled the perforin content of bulk *ex vivo* CD8*^+^* T cells from nine treatment naïve HIV-infected progressors, using the two different anti-perforin antibodies; δG9 which detects the presence of stored or granule-associated perforin and B-D48, which detects granule-associated plus newly formed perforin within cells [29]. A representative experiment from one individual is shown in Fig. 2*a*, with summary data of all individuals in Fig. 2*c*. We found that Tim-3 expressing CD8^+^ T cells contained significantly greater amounts of perforin as measured by δG9 and B-D48. (For δG9, mean expression 30%±3 s.e. versus 22%±4, for Tim 3^+^ and Tim-3**^−^**, respectively and for B-D48, mean expression 40%±2 versus 28%±3, for Tim 3^+^ and Tim-3^–^, respectively). We also examined the correlation between Tim-3 and perforin expression in memory subsets of CD8^+^ T cells by examining CD45RA- memory T cells [39] which will gate out the naïve CD8^+^ T cell populations (for δG9, mean expression 47%±5 s.e. versus 30%±4, for Tim 3^+^ and Tim-3**^−^**, respectively and for B-D48, mean expression 61%±2 versus 43%±3, for Tim 3^+^ and Tim-3**^−^**, respectively). Since δG9 expression is higher in Tim-3 expressing cells, the latter express more granule-associated (or stored) perforin. Since the differences in B-D48 expression between Tim-3 negative and positive cells was similar to that detected by δG9, we assume that the majority of excess perforin expression found in Tim-3 expressing cells is likely due to stored or granule associated perforin. These observations suggest that the higher frequency of perforin expression in Tim-3 expressing cells is due primarily to granule-associated perforin. We then speculated that this higher perforin content, which is mainly of the stored conformation, is due to a lower ability of CD8^+^ T cells to release perforin from its granular stores. This led us to further investigate the ability of CD8^+^ T cells to release their perforin.

**Figure 6:**
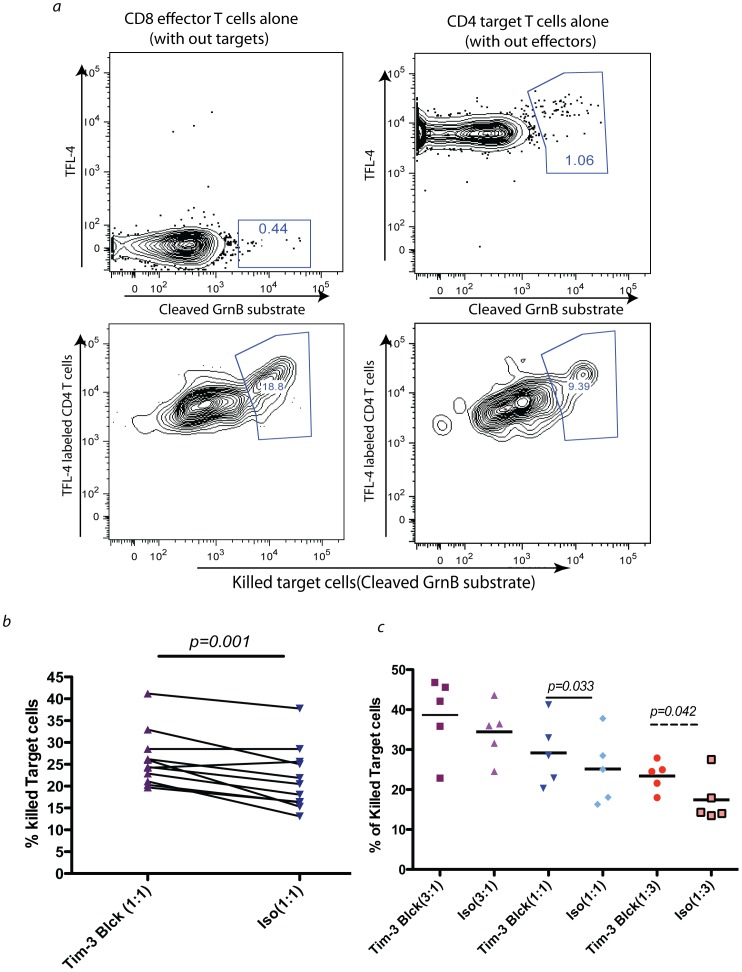
Blocking the Tim-3 signalling pathway enhances the *per cell* cytotoxicity of CD8^+^ T cells. CD4^+^ T cells from chronically HIV infected individuals were transfected with HIV *gag* mRNA. These target cells were labeled with TFL-4 after transfection and then co-incubated with autologous CD8^+^ T cells. Granzyme B substrate is added to cell culture. Cytotoxicity of target cells is determined by the presence of activated GrnB substrate found within the target cells. Experiments are performed either in presence of 10 µg/ml 2E2 Tim-3 blocking or isotype control antibodies. In *a*) is one representative experiment showing the percentage of killed target cells (TFL-4^+^ and cleaved GrnB substrate^+^) in the presence of 2E2 blocking antibody and isotype control antibody respectively in 1∶1 ratio of effectors to target cells. Negative controls were shown in top two plots for either CD8^+^ or CD4^+^ T cells. In *b*), are summary of results from 11 different subjects, each solid dot represents the average of two independent experiments from each individual. In *c*), shown are summary data for five different individuals in three different ratios of effector to target cells (3∶1,1∶1,1∶3) (*p* value based on two tailed paired t test).

### Tim-3 Expressing CD8^+^ T Cells are Exhausted in Terms of Degranulation Capacities

The degranulation ability of CD8^+^ T cells was examined by measuring the surface expression of lysosomal-associated membrane protein 1 (LAMP1, or CD107a) during stimulation with cognate antigen. *Ex vivo* PBMC from chronically HIV infected individuals were stimulated with a pool of HIV Gag peptides for 6 hours while stained for the degranulation marker CD107a [35] and assessed for Tim-3 expression, as shown in Figure 3*a*. Tim-3 positive CD8^+^ T cells were shown not to release their granules as effectively as their Tim-3 negative counterparts after peptide stimulation (Fig. 3*b*) as demonstrated by reduced CD107a expression during peptide stimulation. These findings suggest that this lower degranulation ability may be responsible for the higher accumulation of perforin inside the cells. In order to check if this incapacity for degranulation is HIV specific or a general defect in Tim-3^+^ T cells, we also stimulated *ex vivo* PBMCs from chronically HIV infected individuals with SEB, which is a general non-specific mitogen. We found lower CD107a expression and hence degranulation on Tim-3^+^ subsets after SEB stimulation (Figure 3*a and c*). It is possible that HIV antigen specific cells were enriched in the Tim-3 negative fraction, which could induce greater degranulation after peptide stimulation, however, when PBMCs were stained for HIV specific tetramers containing epitopes that were also included in the peptide pool, we found the frequency of antigen specific cells tended to be more enriched in the Tim-3 positive population and as Tim-3 expression is stable or even increases to some extent after six hours of stimulation, it indicates an even distribution of HIV specific cells after stimulation (Figure S1).

### Blocking Tim-3 Pathway Enhances the Release of Perforin

To test the effect of Tim-3 pathway blocking on the release of perforin, we employed a perforin release ELISA experiment. We sorted *ex vivo* CD8^+^ T cells from 5 untreated chronically HIV infected individuals and stimulated them with a HIV Gag peptide pool for 6 hours in the presence of either 10 µg/ml Tim-3 antagonistic 2E2 antibody or an isotype control antibody. Perforin released in the supernatant at 6 hours was measured by ELISA. As is shown in Figure 4*a*, we found that Tim-3 blocking enhances the release of perforin in supernatants from HIV-specific CD8^+^ T cells (Fig. 4*a*, Mean ± SD concentration of perforin (pg/ml) 191.6±43.11 vs. 163.2±36.83 for Gag peptide stimulation in the presence of anti-Tim-3 antibody or control respectively-P = 0.007). Interestingly, blocking Tim-3 also enhanced spontaneous perforin release from *ex vivo* samples (Fig 4 *a,b medium*). We also examined the release of perforin after Tim-3 pathway blocking in CD8^+^ T cells from four chronically HIV infected, untreated, viral controllers (VC), and found variable responses, with enhancement in 2/4 (Fig. 4b). We also examined ex vivo CD8^+^ T cells directed against other viruses. For example, CD8^+^ T cells from two HCV mono-infected and three CMV infected HIV negative individuals were stimulated with HCV NS3 or CMV pp65 peptides, respectively, and perforin release was examined in the presence of Tim-3 blockade or Isotype. Again, we observed variable levels of enhancement of perforin release in these individuals (data not shown) indicating that Tim-3 blockade will not always enhance perforin release of virus specific cells.

**Figure 7:**
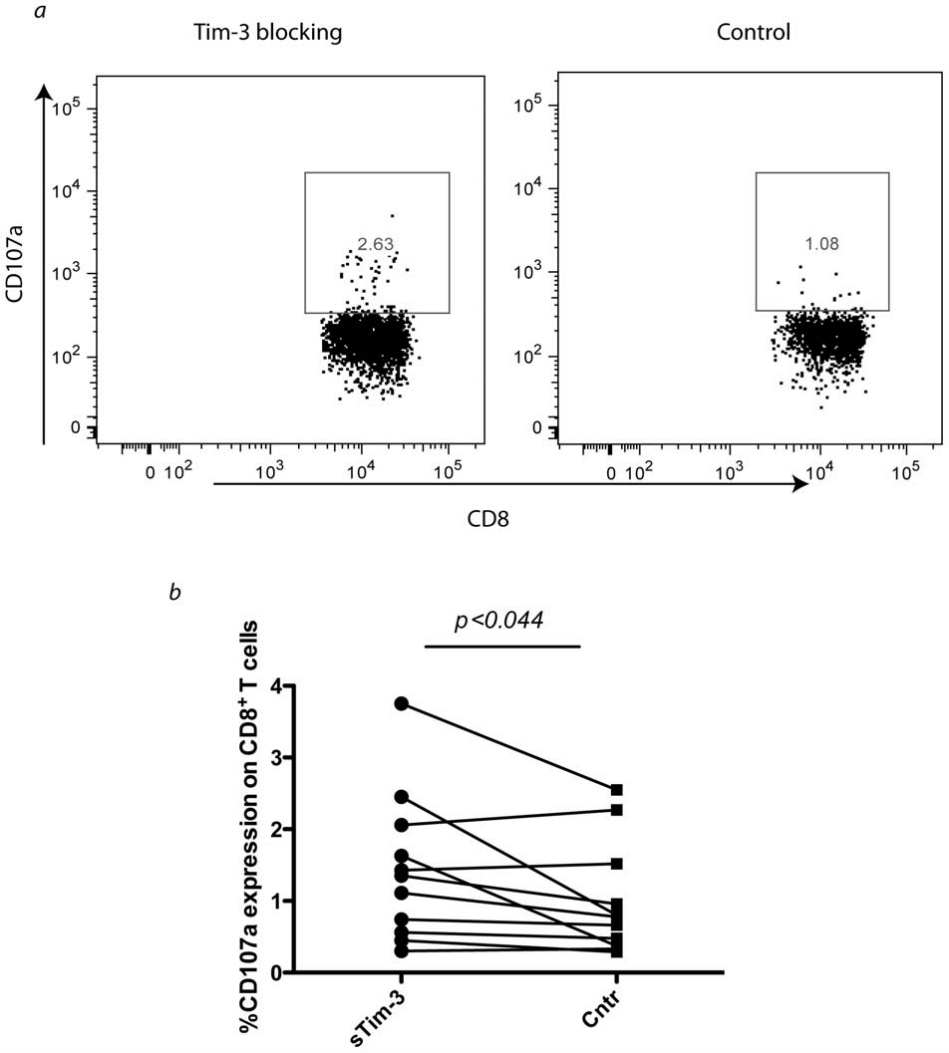
Tim-3 pathway blocking increases the ability of CD8^+^ T cells to degranulate upon further in vitro re-stimulation. *Ex vivo* PBMC from HIV infected subjects were stimulated for 6 hours with a pool of HIV Gag peptides in the presence of sTim-3 (final concentration of 2 µg/ml) or just medium alone, and CD107a expression was measured. In a), a representative experiment showing CD107a expression with or without sTim-3 added to the cell culture from the same individual, In b), are summary of data for all 11 subjects (*p* value based on two tailed paired t test).

### Blocking the Tim-3 Pathway Enhances the Ability of CD8^+^ T Cells to Suppress HIV Infection of Autologous CD4^+^ T Cells

In order to examine the cytotoxic activity of Tim-3 expressing CD8^+^ T cells, we first used a suppression assay that utilizes autologous HIV-infected CD4^+^ T cells. We tested the overall ability of CD8^+^ T cells to kill HIV infected autologous CD4^+^ T cells with or without Tim-3 pathway blocking. We negatively sorted CD4^+^ T cells from chronically HIV infected subjects and infected the cells with a primary HIV virus ((US1/GS 004(91US-1)-CCR5 HIV-NIH AIDS Research and Reference Reagent Program) since the levels of endogenous virus infection of autologous CD4^+^ target cells is <1%. We usually reached an average of 30–40% of infected CD4^+^ target cells in our control samples (without CD8^+^ T cells) at day three. At the same time, we sorted CD8^+^ T cells from the same individual and co-cultured them with anti-Tim-3 antibody (Clone 2E2) or an isotype control antibody (IgG1) for one day. We then combined the CD4^+^ and CD8^+^ T cells at different ratios for 3 days to suppress the infection. We observed significantly higher levels of cytotoxicity after Tim-3 pathway blocking, evidenced by reduced number of infected CD4^+^ T cells remaining in the co-culture following Tim-3 blockade (Fig 5*c–e*, M ± SD = 7.0%±4.0% vs. 18.8% ±8.6% for 2E2 mAb and control Ab respectively P = 0.043). It is possible that Tim-3 blockade may have improved the survival of CD8^+^ T cells, which could explain their enhanced cytotoxicity; however, we did not observe significant differences of CD8^+^ T cell numbers in isotype versus Tim-3 antibody conditions at the end of each experiment (Figure S2). We also observed a similar effect at other effector (CD8): target (CD4) ratios that we used (1∶16, 1∶4) (data not shown). As expected [34], CD8^+^ T cells from untreated viral controllers (plasma viral load<50 copies/ml) potently suppressed virus compared to chronic progressors (Fig. 5d and f), as well, their CD8^+^ T cells do not express high levels of Tim-3 (not shown), and we saw minimal enhancement of suppression with Tim-3 blockade (Fig. 5f). In addition, Tim-3 blockade had no effect on infected CD4^+^ T cells in the absence of autologous CD8^+^ T cells (Fig 5*a*). We also observed that CD8^+^ T cells from HIV un-infected individuals do not suppress autologous *in vitro* infected CD4^+^ T cells even in the presence of Tim-3 blocking antibodies, further indicating the indispensible role of TCR engagement by antigen in infection suppression (Fig. 5*b*).

### Blocking the Tim-3 Pathway Enhances the Direct Cytotoxicity of CD8^+^ T Cells

Finally, we examined CD8^+^ T cells cytotoxicity on a *per-cell* basis by using a newly developed protocol of granzyme B associated cell cytotoxicity assay [38]. We studied *ex vivo* samples from 11 untreated HIV-infected progressors. In this assay, autologous target CD4^+^ T cells are transfected with a HIV *gag* mRNA which on average had a transfection efficiency of 20–30% (assessed by staining for a HIV gag p24 antigen by flow cytometry, not shown). Negatively sorted CD4^+^ T cells that were transfected with HIV *gag* mRNA and labeled with TFL-4 dye, were co-cultured with autologous CD8^+^ T cells in the presence of blocking Tim-3 Ab or isotype for an hour. The level of cleaved granzyme B substrate inside target CD4^+^ T cells was then assessed by flow cytometry (See methods and materials). Representative data from one individual along with negative controls for the dyes that were used, are shown in Figure 6a. Summary data of 11 individuals using 1∶1 ratio of CD4:CD8 are shown in Figure 6b and summary data for 5 individuals with other ratios are shown in Figure 6c. These experiments demonstrate that blocking Tim-3 enhances the ability of HIV-specific CD8^+^ T cells to deliver granzyme B to target CD4^+^ T cells.

### Blocking the Tim-3 Pathway Enhances the Ability of HIV-specific CD8^+^ T Cells to Degranulate

To further confirm our hypothesis which Tim-3 pathway blocking leads to higher perforin release and consequently higher cytotoxicity, we investigated the effects of Tim-3 pathway blocking on degranulation ability of CD8^+^ T cells. *Ex vivo* PBMC from untreated chronically HIV infected individuals were subject to 6 h stimulation with pool of HIV Gag peptides (2 µg/ml/peptide). We then examined the CD107a expression of total CD8^+^ T cells in the presence of sTim-3 (2 µg/ml final concentration) used to block binding of Tim-3 on T cells to its ligands or a medium control. As is shown in Fig. 7a and 7b, Tim-3 pathway blocking enhances the ability of CD8^+^ T cells to degranulate as is evident by higher CD107a expression upon peptide stimulation. This finding again confirms this idea that Tim-3 pathway blocking, mainly through enhancement of degranulation and release of perforin and granzymes, leads to higher cytotoxicity in antigen-specific CD8^+^ T cells.

## Discussion

In this work, for the first time we showed that Tim-3 expression dampens the direct cytotoxicity of CD8^+^ T cells. As it has been previously shown in mice and human studies, Tim-3 positive T cells are dysfunctional in terms of cytokine production or proliferation [15], [40], [41], [42], [43], [44], [45]. They are also more susceptible to apoptosis [46]. It is now widely accepted that direct cytotoxicity of T cells is most important in controlling HIV infection [1], [12], [23]. One of the main molecules that has an indispensible role in eradication of viral infections is perforin. Perforin is stored inside the secretory granules of cytotoxic lymphocytes such as CD8^+^ T cells and NK cells [12], [22], [23], [47], [48], [49], [50], [51], [52], [53], [54], [55], [56], [57]. Having the same crucial role in HIV infection [48], [58], [59], [60], our primary objective in this study was to examine the perforin content and hence cytotoxicity of CD8^+^ T cells based on their Tim-3 phenotypic characteristics. We found that Tim-3 positive CD8^+^ T cells have higher amounts of perforin stored as granules, that is in contrast to the lower perforin content of PD-1 expressing cells as has previously been shown by others [61] and also in our lab (data not shown). We also found higher perforin content in Tim-3^+^ antigen specific cells. These cells were also high expressors of T-bet consistent with the role of T-bet in promoting perforin production. These features regarding Tim-3 were not only restricted to HIV specific T cells as we also have found similar findings in CMV specific cells (data not shown), however, CMV specific T cells exhibit much lower Tim-3 expression than HIV- specific CD8^+^ T cells [15]. Although it was initially surprising to us that a population of T cells, which are considered exhausted and dysfunctional, have higher *immediate* abilities to kill their target cells, based on the staining patterns of the two perforin antibodies used, we propose that the increased perforin levels associated with Tim-3 was predominantly granule-associated. This finding, together with the fact that Tim-3 positive cells are dysfunctional in their abilities to release their secretory granules (as is measured by CD107a expression), further consolidates the idea that Tim-3 receptor binding to its ligand(s), prevents the cells from releasing their perforin and therefore perforin builds up in their granules resulting in higher stored perforin in these cells. The higher perforin release that we observed in our perforin ELISA experiments after blocking Tim-3 pathway signalling further supports this hypothesis.

We also measured the direct cytotoxicity of CD8^+^ T cells in the context of HIV infection. We clearly showed that with blocking the Tim-3 pathway we can get higher eradication of infected target cells by autologous CD8^+^ T cells showing an enhanced overall cytotoxicity by blocking Tim-3 receptors. We also showed significant differences in killing activity with Tim-3 blockade using a granzyme release assay further narrowing down this increased overall killing activity to the ability of CD8^+^ T cells to release perforin and granzyme. Although, the differences in direct cytotoxcity as determined by perforin release (Fig. 4), granzyme release (Fig. 6), and CD107a surface expression (Fig. 7) appeared to be rather modest with Tim-3 blockade, we predict that small incremental differences in killing activity over one hour would likely be magnified after multiple rounds of killing target cells over 24–48 hours, as manifested by the more substantial reduction in HIV-infected target cells with Tim-3 blockade in three day cultures (Fig. 5).

Thus, our findings further support that the Tim-3 receptor is an inhibitory molecule that tends to dampen the general activation of antigen-specific T cells after an immune reaction peaks and leads to clearance of the infection or tolerance of the antigen [43], [44], [62], [63], [64], [65], [66]. CD8^+^ T cells play a crucial role especially early on the course of HIV infection [60], [67], [68], [69], [70], [71], [72], [73] and the main mechanism that these cells exert their cytotoxic function is through production and release of killing molecules (perforin, granzymes, granulysin). As rapid expression of perforin is considered as a novel correlate of control of HIV replication [23], this novel finding that blocking Tim-3 pathway leads to an increase in perforin release and direct cytotoxicity in particular, further indicates that the Tim-3 pathway might be a potential therapeutic target for the rescue of dysfunctional CD8^+^ T cells resulting in the better suppression of HIV infection.

## Supporting Information

Figure S1 **HIV tetramer specific CD8^+^ T cells is evenly distributed between Tim-3^+^ and Tim-3^−^ T cells.** *Ex vivo* PBMC from HIV chronically infected subjects were stained with a HIV Gag SL9 tetramer and then further stained for Tim-3. In *a), a* representative experiment showing percentage of SL9 specific T cells in Tim-3^+^ and Tim-3**^−^** subpopulations of Total CD8^+^ T cells. In *b*), summary of all data for 5 chronically HIV infected individuals showing higher Tim-3 expression on SL9 specific CD8^+^ T cells compared to Total CD8^+^ T cells. In *c*) summary of all data for 6 chronically HIV infected individuals when stained for a pool of HIV tetramers (pool of 5 different HIV tetramers) showing almost even distribution of HIV specific T cells between these two populations. ns  =  non significant.
(TIF)

Figure S2 **Better cytotoxicity achieved after Tim-3 pathway blocking is not due to better survival of CD8^+^ T cells**. Mean number of CD8^+^ T cells/experiment at the end of three-day culture in the presence or absence of Tim-3 pathway blocking is counted. Shown are summary data from 4 experiments performed in triplicate. Bar  =  standard error. The ratio of CD4:CD8 T cells also remained relatively constant in each individual in two conditions (data not shown) (2E2: Tim-3 pathway blocking antibody-Iso: Isotype control antibody).
(TIF)
