# Supplementary figures and images for: Tim-3 Negatively Regulates Cytotoxicity in Exhausted CD8+ T Cells in HIV Infection
Source: PLoS One. 2012 Jul 5;7(7):e40146. doi: 10.1371/journal.pone.0040146 (PMC3390352; doi:10.1371/journal.pone.0040146)

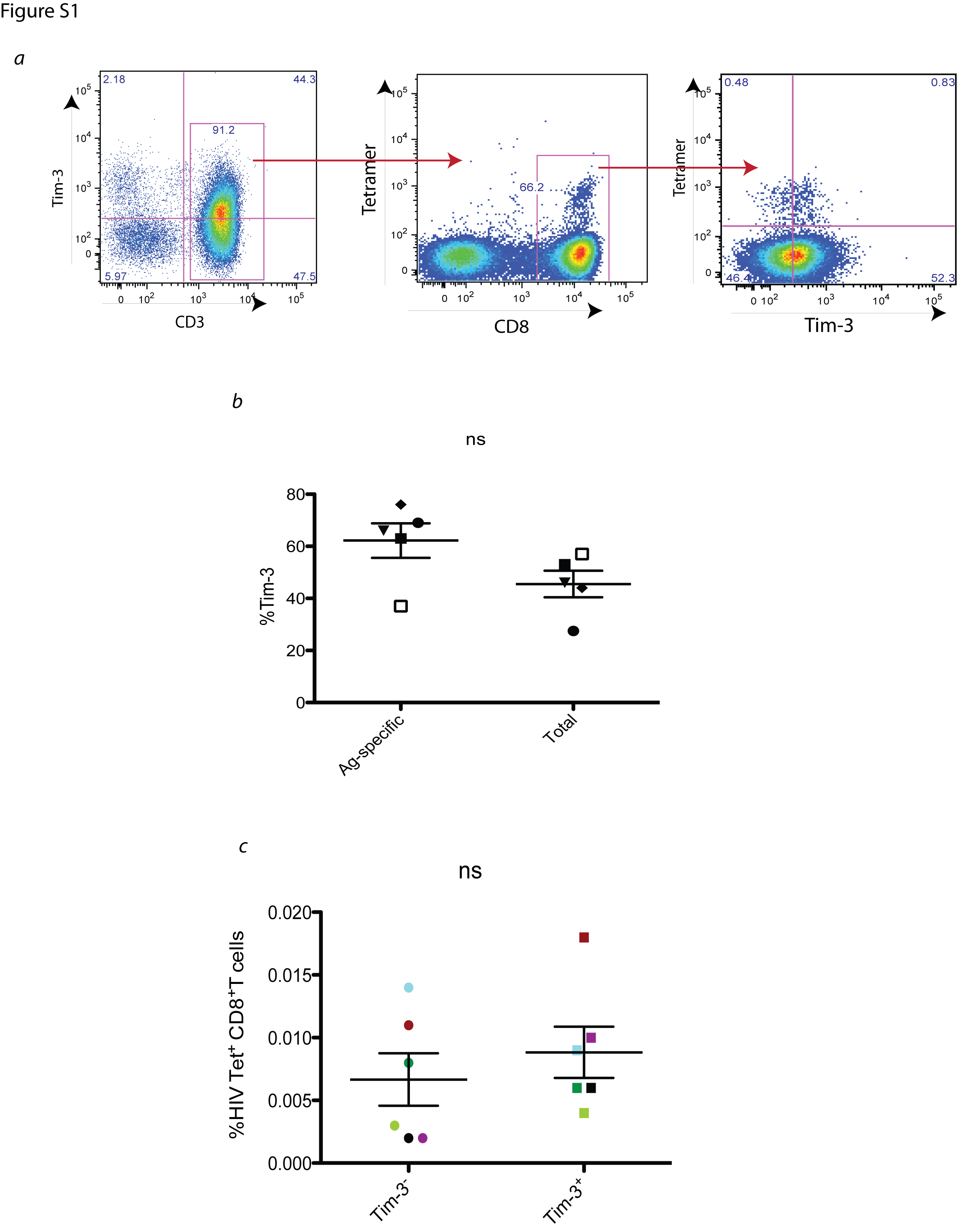

Supplement: Figure S1 — HIV tetramer specific CD8+ T cells is evenly distributed between Tim-3+ and Tim-3− T cells. Ex vivo PBMC from HIV chronically infected subjects were stained with a HIV Gag SL9 tetramer and then further stained for Tim-3. In a), a representative experiment showing percentage of SL9 specific T cells in Tim-3+ and Tim-3− subpopulations of Total CD8+ T cells. In b), summary of all data for 5 chronically HIV infected individuals showing higher Tim-3 expression on SL9 specific CD8+ T cells compared to Total CD8+ T cells. In c) summary of all data for 6 chronically HIV infected individuals when stained for a pool of HIV tetramers (pool of 5 different HIV tetramers) showing almost even distribution of HIV specific T cells between these two populations. ns = non significant. (TIF) [file pone.0040146.s001.tif]

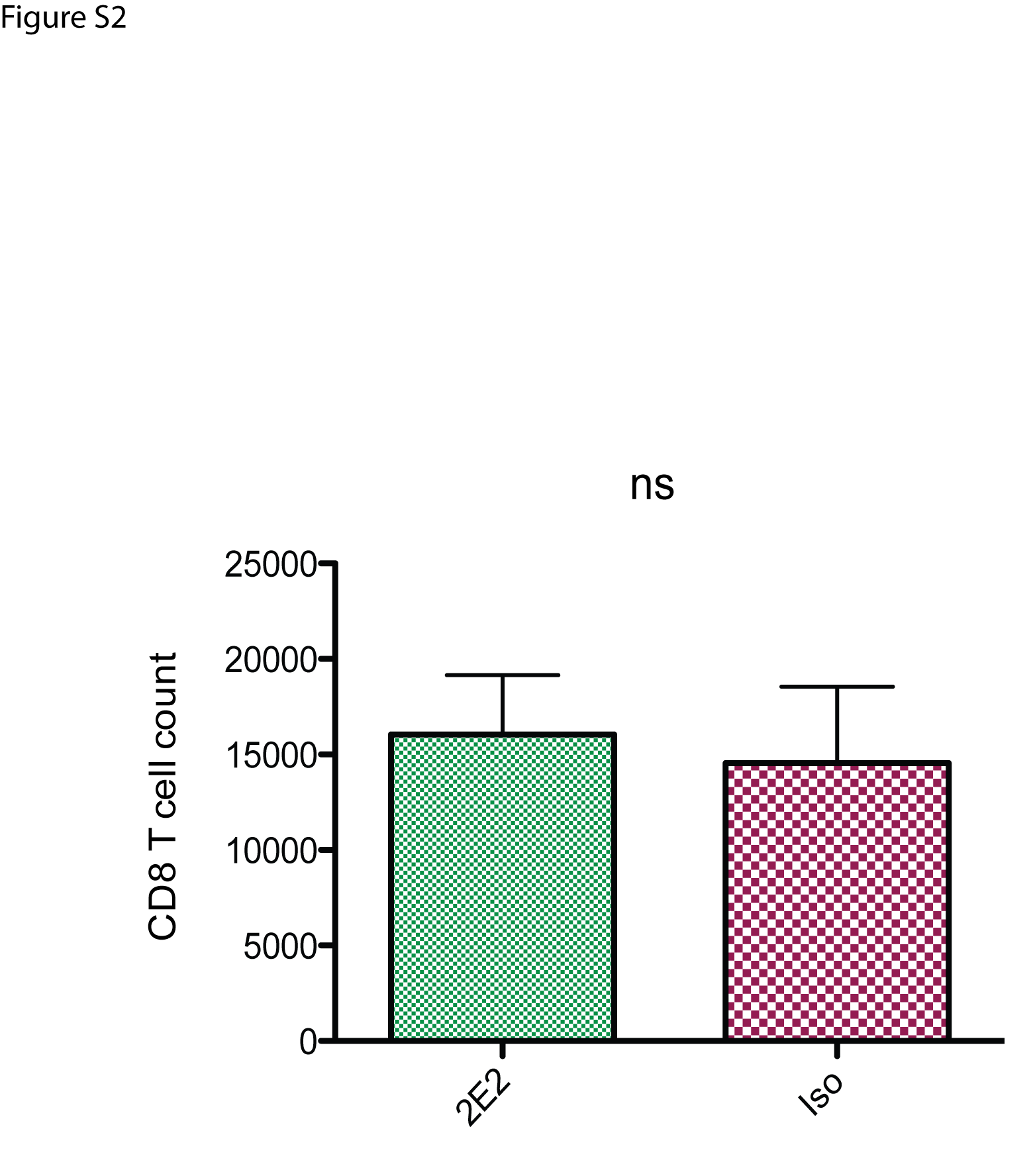

Supplement: Figure S2 — Better cytotoxicity achieved after Tim-3 pathway blocking is not due to better survival of CD8+ T cells. Mean number of CD8+ T cells/experiment at the end of three-day culture in the presence or absence of Tim-3 pathway blocking is counted. Shown are summary data from 4 experiments performed in triplicate. Bar = standard error. The ratio of CD4:CD8 T cells also remained relatively constant in each individual in two conditions (data not shown) (2E2: Tim-3 pathway blocking antibody-Iso: Isotype control antibody). (TIF) [file pone.0040146.s002.tif]
